# Protein disulphide isomerase (PDI) is protective against amyotrophic lateral sclerosis (ALS)-related mutant Fused in Sarcoma (FUS) in in vitro models

**DOI:** 10.1038/s41598-021-96181-2

**Published:** 2021-09-02

**Authors:** S. Parakh, E. R. Perri, M. Vidal, J. Sultana, S. Shadfar, P. Mehta, A. Konopka, C. J. Thomas, D. M. Spencer, J. D. Atkin

**Affiliations:** 1 Macquarie Centre for MND Research, Department of Biomedical Sciences, Faculty of Medicine and Health Sciences, Macquarie University, Sydney, NSW 2109 Australia; 2 Department of Physiology, Anatomy and Microbiology, La Trobe University, Melbourne, VIC 3086 Australia; 3 Department of Biochemistry and Genetics, La Trobe Institute for Molecular Science, La Trobe University, Melbourne, VIC 3086 Australia

**Keywords:** Chaperones, Amyotrophic lateral sclerosis

## Abstract

Mutations in *Fused in Sarcoma (FUS)* are present in familial and sporadic cases of amyotrophic lateral sclerosis (ALS) and frontotemporal dementia (FTD). FUS is localised in the nucleus where it has important functions in DNA repair. However, in ALS/FTD, mutant FUS mislocalises from the nucleus to the cytoplasm where it forms inclusions, a key pathological hallmark of neurodegeneration. Mutant FUS also inhibits protein import into the nucleus, resulting in defects in nucleocytoplasmic transport. Fragmentation of the neuronal Golgi apparatus, induction of endoplasmic reticulum (ER) stress, and inhibition of ER-Golgi trafficking are also associated with mutant FUS misfolding in ALS. Protein disulphide isomerase (PDI) is an ER chaperone previously shown to be protective against misfolding associated with mutant superoxide dismutase 1 (SOD1) and TAR DNA-binding protein-43 (TDP-43) in cellular and zebrafish models. However, a protective role against mutant FUS in ALS has not been previously described. In this study, we demonstrate that PDI is protective against mutant FUS. In neuronal cell line and primary cultures, PDI restores defects in nuclear import, prevents the formation of mutant FUS inclusions, inhibits Golgi fragmentation, ER stress, ER-Golgi transport defects, and apoptosis. These findings imply that PDI is a new therapeutic target in FUS-associated ALS.

## Introduction

Amyotrophic lateral sclerosis (ALS) is a fatal, adult-onset neurodegenerative disease, and pathological forms of transactive response DNA-binding protein-43 (TDP-43) are the hallmark of almost all ALS patients (97%). Fused in Sarcoma (FUS)/translocated in liposarcoma (TLS) is a member of the FET protein family^1^ that is closely related structurally, functionally, and pathologically to TDP-43. Both FUS and TDP-43 are important regulators of RNA metabolism^2^ and DNA repair^3,4^. Whilst mutations in FUS account for ~ 5% of familial ALS (fALS, 10% of ALS overall) and 1% of sporadic ALS (sALS) cases^5,6^, mutant FUS-ALS is associated with juvenile-onset ALS and a particularly aggressive disease course^7,8^. Frontotemporal dementia (FTD) is related genetically and pathologically to ALS, and FUS inclusions are also present in patients with Frontotemporal lobar degeneration (FTLD) and hereditary essential tremors^9,10^. Furthermore, FUS immunoreactivity has been detected in cells with expanded poly-glutamine protein nuclear aggregates^11^. FUS−/− mice display abnormal neuronal morphology and are not viable due to disruption of immune cell development and genomic instability^2,12^. Together these reports highlight the importance of FUS in normal cellular function, ALS and neurodegeneration more broadly. Both loss of the normal function of FUS and gain^13^ of toxic function are implicated in pathogenesis^14^.

FUS is normally present in the nucleus, but in ALS/FTD it misfolds in the cytoplasm and forms inclusions in both neurons and glia^5,6^. Importantly, FUS immunoreactivity has been detected in inclusions of sALS patient spinal cords^15,16^. Moreover, recently the mislocalisation of FUS to the cytoplasm was proposed to be a central pathological feature of sALS, similar to TDP-43^17^. Over 50 mutations in FUS have been identified in fALS, most of which cluster around its C-terminus (residues 515–524)^7^. The nucleus and defects in nucleocytoplasmic transport are increasingly implicated as an important site of toxicity in ALS. Moreover, several studies have shown that the presence of ALS FUS mutations prevents the nuclear import of FUS, resulting in its mislocalisation to the cytosol^18,19^.

The endoplasmic reticulum (ER) is another important cellular location associated with ALS/FTD, and following expression of mutant FUS in vitro, the accumulation of misfolded proteins in the ER leads to ER stress and induction of the unfolded protein response (UPR)^20^. Mutant FUS also inhibits protein transport from the ER to the Golgi apparatus in neuronal cells^21^. Specific morphological characteristics of the Golgi are required for maintaining its optimal function, and fragmentation of the Golgi results from inhibition of ER-Golgi transport. Moreover, fragmentation of the neuronal Golgi is widely described in ALS and is triggered following expression of mutant FUS^22^.

Protein disulphide isomerase A1 (PDIA1, henceforth designated as ‘PDI’) is the prototype of an extended family of chaperones localised primarily in the ER. Whilst PDI proteins possess general chaperone activity and thus prevent protein misfolding and aggregation^23^, they also mediate disulphide bond formation in proteins by oxidoreductase activity. Mutations in both PDI and closely related family member ERp57^24–26^ are implicated as genetic risk factors for ALS^27^, are associated with loss of oxidoreductase activity^24^. Moreover, both proteins are protective against typical cellular ALS phenotypes induced by misfolded, mutant forms of SOD1 and TDP-43, against motor dysfunction and axonopathy in zebrafish mutant SOD1 models^24^, and ERp57 is protective against motor impairment and early denervation in SOD1^G93A^ mice^28^. However, it remains unknown whether PDI is protective against ALS phenotypes associated with mutant FUS. Therefore, in this study we investigated this possibility. We demonstrate in neuronal cells expressing mutant FUS that PDI is protective against inclusion formation, inhibition of both nuclear import and ER-Golgi transport, Golgi fragmentation, ER stress and apoptosis. These findings therefore imply that PDI is a novel therapeutic target for FUS-associated ALS.

## Results

### Co-expression of PDI is protective against mutant FUS induced inclusion formation in a neuronal cell line and primary cells

**Figure 1:**
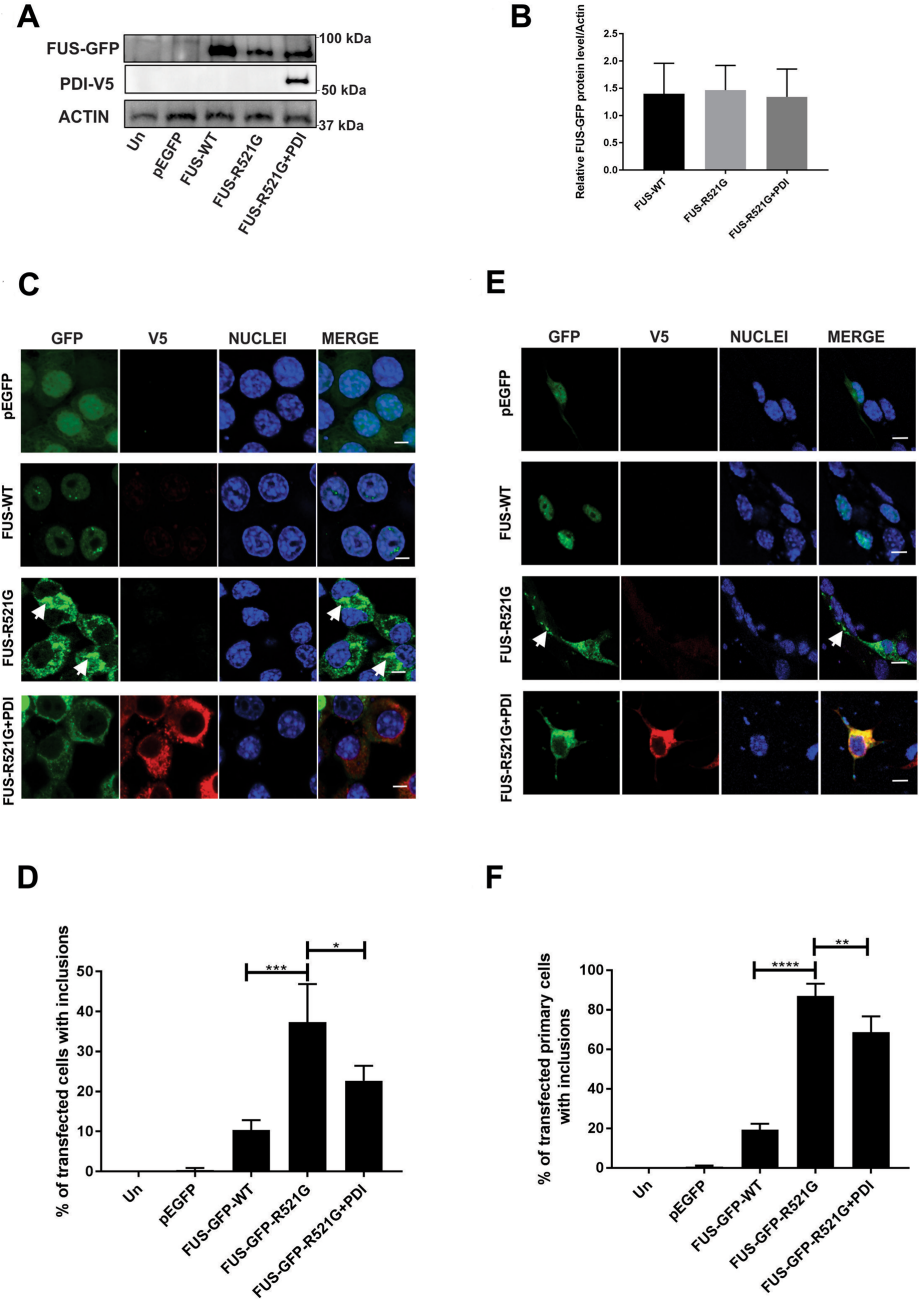
Over-expression of PDI is protective against mutant FUS- induced inclusion formation in a neuronal cell line and primary cultures. (**A**) Western blotting of cell lysates, in which wild-type FUS-GFP (FUS-WT) was expressed with empty vector pcDNA3.1 and mutant FUS-GFP-R521G was expressed with either empty vector pcDNA3.1 or PDI-V5; untransfected (Un) cells are represented in the first lane. The blots were probed with anti-V5 antibody to confirm the presence of PDI, and reprobed with anti-FUS antibody and anti-actin as a loading control. Approximate molecular weight markers in kilodaltons are shown on the right. Whole blots showing the position of molecular weight marker bands are represented in supplementary Figure S1. (**B**) Densitometric quantitation of FUS protein levels normalized to actin from the immunoblots shown in (**A**) confirms similar transfection efficiency in each population and that similar amounts of each protein were expressed. (**C**) Immunofluorescence of EGFP in cells expressing EGFP (row 1), FUS-GFP-WT (row 2) or mutant FUS-GFP-R521G with empty vector (row 3, inclusions > 1 µm, highlighted by white arrows), co-expressed with PDI (row 4), 72 h post-transfection. Scale bar = 5 µm. (**D**) Quantification of cells in (**C**) reveals significantly fewer cells formed inclusions when PDI (*p < 0.05) was co-expressed with FUS-GFP-R521G compared to empty vector. (**E**) Immunofluorescence detection of EGFP-positive inclusions (> 1 µm), present in mouse primary cells co-expressing EGFP only (row 1), FUS-GFP-WT (row 2) or mutant FUS-GFP-R521G with empty vector (row 3), with PDI (row 4). Scale bar = 10 µm. (**F**) Quantification of cells in (**E**) reveals significantly fewer cells formed inclusions when PDI (**p < 0.01) was co-expressed with FUS-GFP-R521G compared to vector only. Values represent mean ± SD, n = 3. ****p < 0.0001, ***p < 0.001, **p < 0.01, *p < 0.05.

Firstly, the effect of PDI on the formation of mutant FUS cytoplasmic inclusions in vitro^18^ was examined, and the ALS-associated R521G mutant was chosen because it is a common and highly pathogenic familial mutation. Wild-type (WT) FUS or mutant R521G tagged with green fluorescent protein (GFP)^29^, or vector pEGFP-N1 only as a control, were co-expressed with PDI tagged with V5^24^ or empty vector (pcDNA3.1), in a neuroblastoma cell line, Neuro-2a. Immunoblotting of cell lysates for V5 and FUS was performed to confirm that similar levels of protein were expressed and of the expected size in each case (Fig. 1A). Quantitation of these immunoblots by densitometry confirmed that over-expression of PDI did not significantly alter the levels of FUS-GFP when normalized to actin (Fig. 1B).

The formation of mutant FUS inclusions was examined by quantifying the percentage of transfected cells bearing large compact GFP-positive aggregates, using confocal fluorescence microscopy (Fig. 1C). Cells expressing EGFP alone formed negligible inclusions (< 1%), as expected. For all the cellular phenotypes examined in this study, expression of FUS-GFP-WT gave very similar results to controls (empty vector and untransfected cells). Hence it was not possible to examine whether PDI was protective against most previously described phenotypes associated with FUS-WT over-expression in disease models, such as inclusion formation here. In the absence of PDI, significantly more cells expressing mutant FUS-GFP-R521G with empty vector pcDNA3.1 (37%, ***p < 0.001) formed inclusions compared to those expressing FUS-GFP-WT, also as expected (10%). However, co-expression of PDI with mutant FUS resulted in significantly fewer inclusions compared to cells transfected with mutant FUS and empty vector (23%, *p < 0.05, Fig. 1D), and this proportion was now similar to FUS-GFP-WT expressing cells. Hence, these data reveal that over-expression of PDI is protective against the formation of mutant FUS inclusions.

To validate these results, mouse cortical primary cultures (containing both neurons and glia) at embryonic day 16–18 were co-transfected as above (Fig. 1E). As expected, few inclusions were formed in EGFP only cells, whereas significantly more cells expressing mutant FUS-GFP-R521G with empty vector (87%, ****p < 0.0001) displayed inclusions compared to those expressing FUS-GFP-WT (20%). However, this proportion was significantly reduced (69%, **p < 0.01) when PDI was co-expressed with mutant FUS (Fig. 1F). Hence, PDI is protective against the formation of mutant FUS inclusions in primary cells, thus confirming the results obtained in Neuro-2a cells.

### Over-expression of PDI is protective against mutant FUS induced nuclear import defects in a neuronal cell line

We next investigated whether PDI is protective against the inhibition of nuclear import induced by mutant FUS. For this purpose, the fluorescent reporter NES-StdTomato-NLS (referred to hereafter as ‘Std-Tomato’), in which StdTomato is flanked by a NES (nuclear export sequence) and a NLS (nuclear localisation sequence), was used. This reporter is widely used to examine nucleocytoplasmic transport because it accumulates in the cytoplasm in cells when the nuclear import machinery is dysregulated^30,31^. Hence, cytoplasmic accumulation of the reporter indicates inhibition of nuclear import. For this purpose, we used a N-terminal tagged FUS construct so that the PY-NLS nuclear targeting signal was retained.

**Figure 2:**
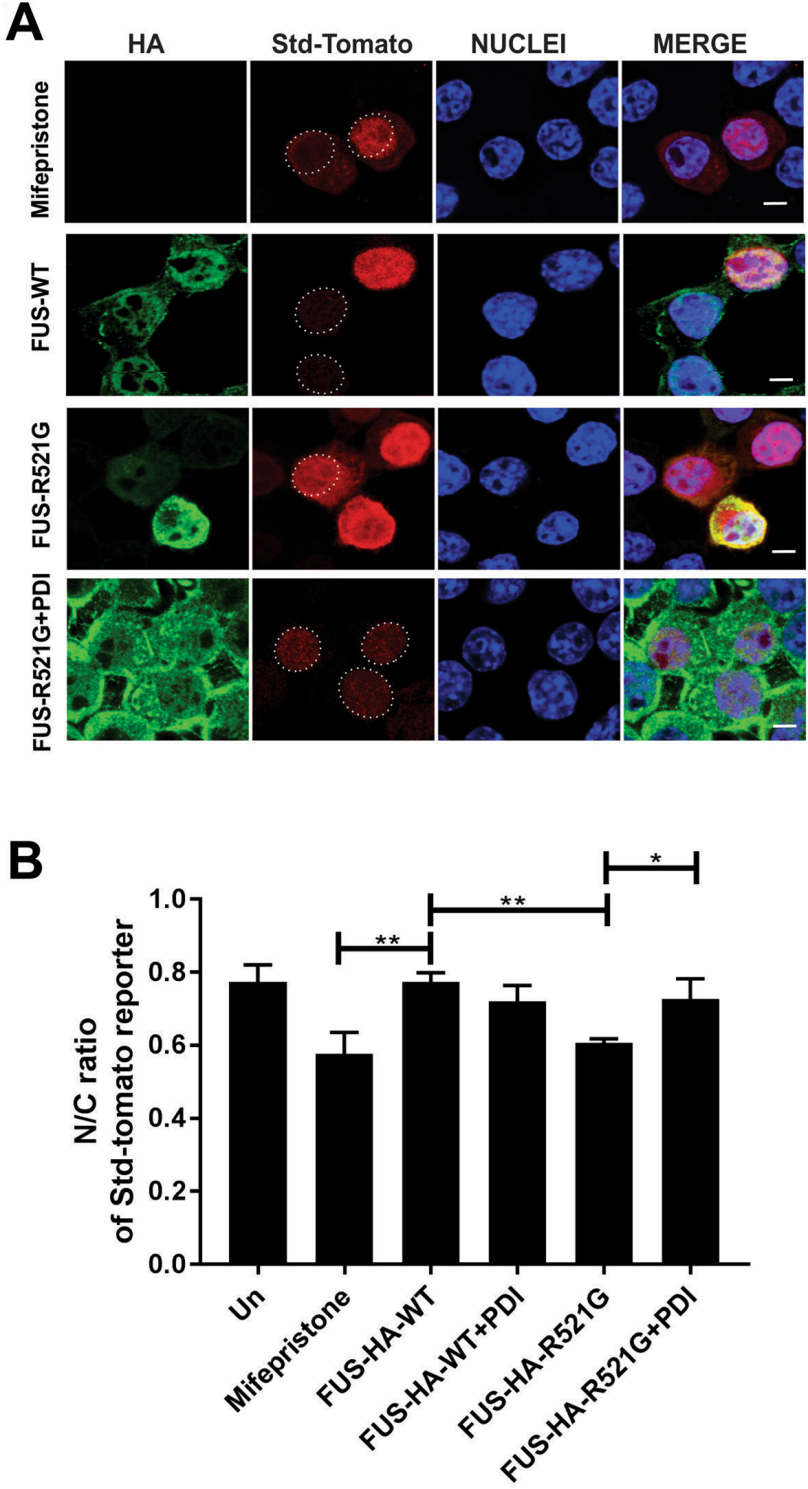
Over-expression of PDI is protective against inhibition of nuclear import induced by mutant FUS in a neuronal cell line. (**A**) Fluorescent microscopy images of cells 48 h post-transfection, expressing FUS-HA, NES-tdTomato-NLS (Std-Tomato) and either PDI or empty vector following immunocytochemistry in Neuro-2a cells. Cells transfected with 1 µm Mifepristone (row 1), FUS-HA-WT (row 2), mutant FUS-HA-R521G with empty vector (row 3) or mutant FUS-HA-R521G co-expressing PDI (row 4). Scale bar = 5 µm. (**B**) Quantification of the nuclear to cytoplasmic (N/C) fluorescence intensity ratio of Std-Tomato in Neuro-2a cells expressing FUS-HA-WT or mutant FUS-HA-R521G or co-expressing PDI in (**A**). Significantly more cytoplasmic reporter is present in cells expressing mutant FUS-HA-R521G (**p < 0.05) compared to FUS-HA-WT. However, co-expression of PDI (*p < 0.05) results in more reporter localised in the nucleus in FUS-HA-R521G cells compared to those expressing empty vector. Values represent mean ± SD, n = 3. **p < 0.01, *p < 0.05.

Neuro-2a cells were co-transfected with Std-Tomato, hemagglutinin (HA)-tagged FUS-WT or mutant FUS-HA-R521G, and either empty vector or V5-tagged PDI (Fig. 2A). The ratio of intensity of the nuclear-to-cytoplasmic (N-to-C) fluorescence of Std-tomato was quantitated using confocal microscopy and ImageJ. In untransfected cells and cells expressing FUS-HA-WT, the N-to-C ratio was 0.77 and 0.77 respectively, revealing that Std-tomato was localised predominately in the nucleus. Hence nuclear import was efficient in these cells. As a positive control we also treated cells with mifepristone a specific inhibitor of importin α/β-mediated nuclear transport which therefore perturbs nuclear import^32^. A significant reduction in the N-to-C fluorescent intensity ratio was detected in cells treated with mifepristone (0.57, **p < 0.01) compared to FUS-HA-WT. Similarly, significant reduction in the N-to-C fluorescent intensity ratio was detected in cells expressing mutant FUS-HA-R521G with empty vector compared to FUS-HA-WT (0.60, **p < 0.01, Fig. 2B). This result indicates that significantly more Std-tomato reporter was present in the cytoplasm^33^ of mutant FUS expressing cells, and hence that nuclear import was inhibited. Interestingly, however, upon expression of PDI, nuclear import was restored in mutant FUS expressing cells (*p < 0.05 vs empty vector), and the N-to-C fluorescent intensity ratio detected was 0.72, similar to cells expressing FUS-WT. These results therefore imply that PDI is protective against inhibition of nuclear import induced by mutant FUS.

### Over-expression of PDI is protective against mutant FUS induced ER-Golgi transport defects in a neuronal cell line

**Figure 3:**
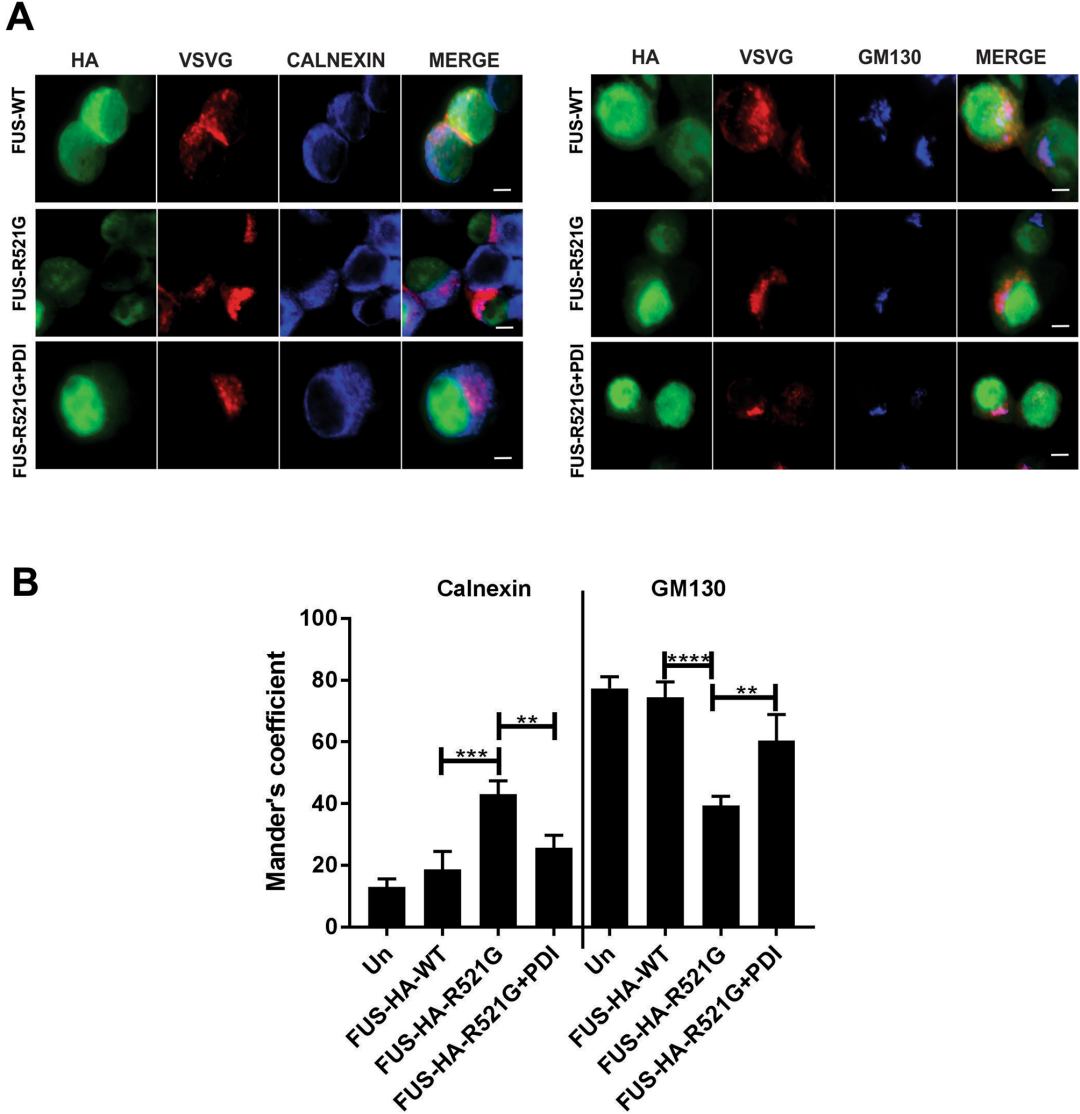
Over-expression of PDI restores ER-Golgi transport in mutant FUS expressing cells. (**A**) Representative fluorescent images of cells co-expressing HA-tagged FUS-WT or FUS-HA-R521G, with VSVGts045-mCherry and either PDI-V5 or empty vector. Immunocytochemistry was performed using antibodies against markers of the ER (calnexin) or Golgi apparatus (GM130). VSVGts045-mCherry was trapped in the ER at 40 °C, cycloheximide was added, and the temperature was shifted to the permissive temperature (32 °C) for 30 min. At 32 °C, VSVGts045 is transported to Golgi and does not colocalise with calnexin in cells expressing FUS-HA-WT (row 1). In comparison, transport is inhibited in mutant FUS-HA-R521G with empty vector expressing cells, where less VSVGts045 was colocalised with GM130 (row 2). However, over-expression of PDI in these cells resulted in more VSVGts045 colocalised with GM130 (row 3). Scale bar = 5 μm. (**B**) Quantification of the degree of co-localization of VSVGts045-mCherry with calnexin or GM130 was quantified using Mander's coefficient of cells in (**A**). Significantly more (***p < 0.001) VSVGts045 was retained in the ER and less in the Golgi in mutant FUS-HA-R521G with empty vector cells compared to FUS-HA-WT (****p < 0.0001). In contrast, significantly more VSVGts045 was present in the Golgi (**p < 0.01) and less in the ER (**p < 0.01) when PDI was co-expressed with mutant FUS compared to cells expressing empty vector with mutant FUS. Values represent mean ± SD, n = 3. ****p < 0.0001, ***p < 0.001, **p < 0.01.

We next investigated whether expression of PDI was protective against inhibition of ER-Golgi transport in cells expressing mutant FUS. For this purpose, we used a temperature sensitive mutant of vesicular stomatitis viral glycoprotein (VSVG^ts045^), a classical ER-Golgi transport marker^21,34^. Neuro-2a cells were co-transfected with mCherry-tagged VSVG^ts045^, FUS-HA-WT or mutant FUS-HA-R521G, and either empty vector or V5-tagged PDI, before being incubated first at 40 °C to accumulate VSVG^ts045^ in the ER, and then at the permissive temperature (32 °C) for 30 min. Immunocytochemistry was then performed using antibodies for markers of the ER (calnexin) or Golgi (GM130), followed by fluorescent microscopy (Fig. 3A). Quantification of the localisation of VSVG^ts045^ in either the ER or Golgi was performed using Mander’s co-efficient, in the range from 0 to 1 representing 0–100% overlapping pixels, as described previously^35^.

In untransfected cells, little VSVG^ts045^ (13%) was retained in the ER and most (77%) was transported to the Golgi apparatus after 30 min at the permissive temperature, demonstrating efficient ER-Golgi transport. Similarly, in cells expressing FUS-HA-WT, little VSVG^ts045^ (20%) was retained in the ER and most was transported to the Golgi (74%). However, in cells expressing mutant FUS-HA-R521G with empty vector, as previous, inhibition of ER-Golgi transport was detected relative to the other cell populations^21^. Significantly more VSVG^ts045^ was retained in the ER (43%, ***p < 0.001) and less was transported to the Golgi (39%, ****p < 0.0001) compared to FUS-HA-WT (Fig. 3B). However, when PDI was co-expressed with mutant FUS-HA-R521G, transport between the ER-Golgi was restored; only 25% (**p < 0.01) of cells displayed VSVG^ts045^ retained in the ER and in 60% (**p < 0.01) of cells, VSVGts045 was transported to Golgi. Therefore, these data reveal that over-expression of PDI rescues inhibition of ER-Golgi transport induced by mutant FUS.

### Over-expression of PDI is protective against mutant FUS induced Golgi fragmentation in a neuronal cell line

**Figure 4:**
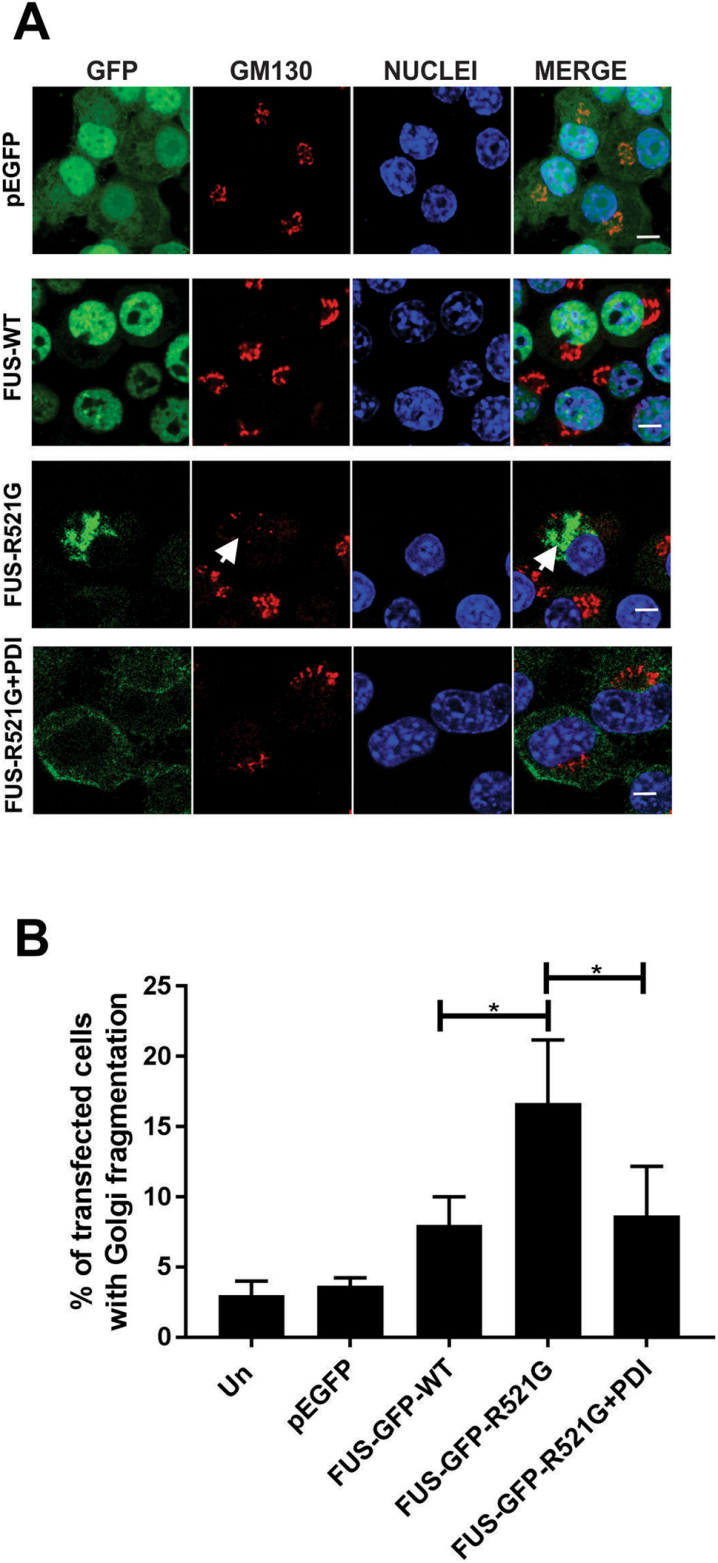
Over-expression of PDI is protective against mutant FUS induced Golgi fragmentation in a neuronal cell line. (**A**) Neuro-2a cells co-expressing EGFP (row 1), FUS-GFP-WT (row 2) or mutant FUS-GFP-R521G with either empty vector, (row 3) or PDI (row 4) at 72 h post-transfection, were subjected to immunocytochemistry for Golgi marker GM130 and counter-staining with Hoechst 33342 stain (nuclei). Most cells expressing EGFP, or FUS-GFP-WT (row 1 and 2) contained an intact Golgi apparatus. Conversely, cells expressing mutant FUS-GFP-R521G with empty vector displayed fragmented Golgi, indicated with white arrows (row 3). However, over-expression of PDI reduced Golgi fragmentation in these cells (row 4). Scale bar = 5 µm. (**B**) Quantification of cells in (**A**). A significant reduction (*p < 0.05) in the proportion of cells with Golgi fragmentation was observed in cells expressing mutant FUS-GFP-R521G with PDI, compared to those expressing mutant FUS with vector only. Values represent mean ± SD, n = 3. *p < 0.05.

We next investigated whether PDI is protective against Golgi fragmentation in cells transfected as above. The morphology of the Golgi apparatus was examined by immunocytochemistry using an anti-GM130 antibody and confocal fluorescence microscopy. The Golgi is normally characterised by continuous stacked membranes, mostly localized to a compact, perinuclear ribbon. In contrast, fragmentation of the Golgi is detected by the presence of punctate structures dispersed throughout the cytoplasm, displaying multiple disconnected elements or tubular-vesicular clusters, as previously described for mutant FUS (Fig. 4A^22^). The Golgi displayed its typical morphology, with continuous staining in a twisted ribbon-like network resembling the Golgi apparatus and little fragmentation, in untransfected cells and those expressing EGFP or FUS-GFP-WT (3%, 4% and 8% respectively). In contrast, in cells expressing mutant FUS-GFP-R521G, the Golgi was markedly different, with discrete tubulovesicular structures that were dispersed into discrete vesicles, consistent with fragmentation. The Golgi apparatus was fragmented in significantly more cells (17%, *p < 0.05) expressing mutant FUS-GFP-R521G with empty vector compared to FUS-GFP-WT (Fig. 4B). However, significantly fewer mutant FUS cells co-expressing PDI displayed fragmented Golgi compared to those expressing mutant FUS with empty vector only (9%, *p < 0.05), which was now not significantly different to controls. Hence, these data reveal that PDI inhibits Golgi-fragmentation induced by mutant FUS.

### Over-expression of PDI is protective against mutant FUS induced ER-stress in a neuronal cell line

**Figure 5:**
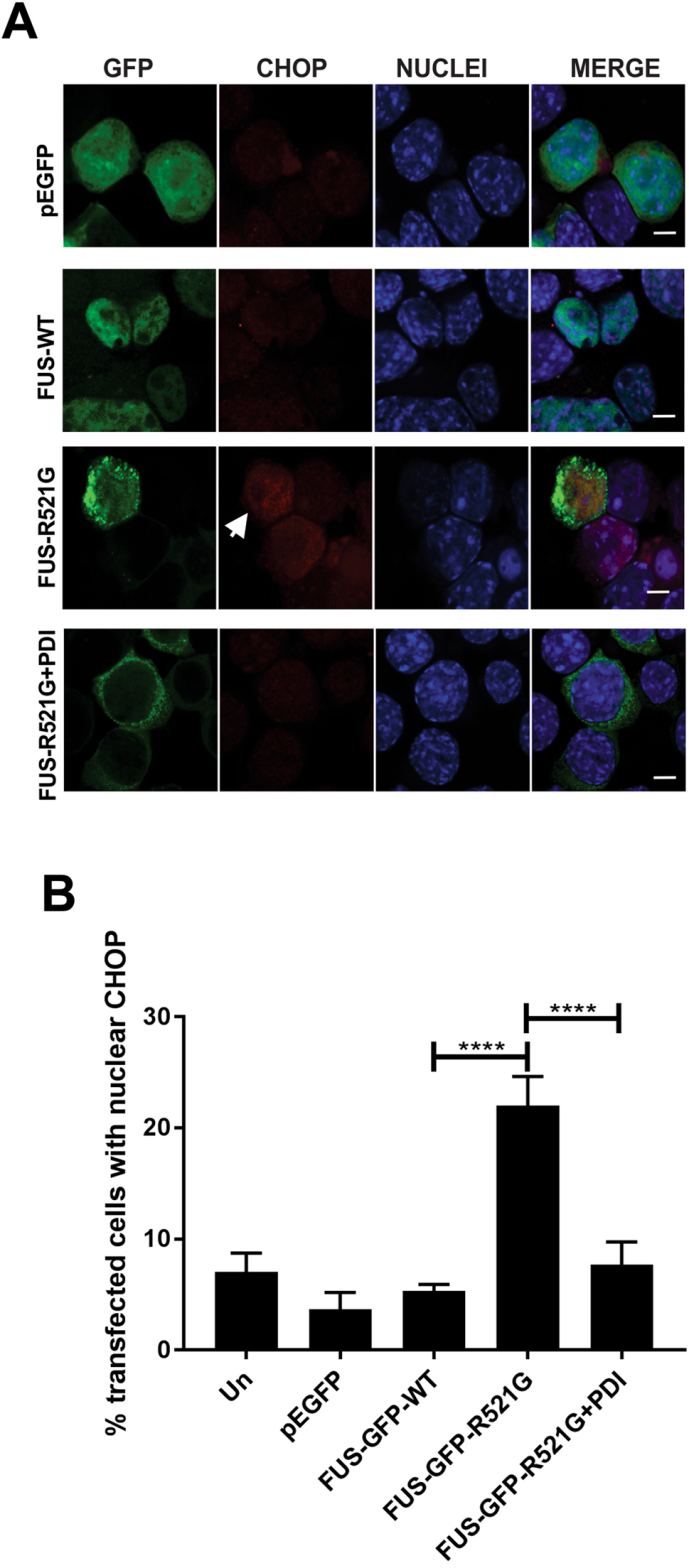
Over-expression of PDI inhibits nuclear immunoreactivity to CHOP, and hence ER stress, in mutant FUS expressing cells. (**A**) Neuro-2a cells co-expressing EGFP (row 1), FUS-GFP-WT (row 2) or mutant FUSGFP-R521G with empty vector, (row 3), or with PDI (row 4) at 72 h post-transfection, were subjected to immunocytochemistry using anti-CHOP antibodies and counter-staining with DAPI. Nuclear immunoreactivity to CHOP was used as a marker of ER-stress, indicated with white arrow. Most untransfected cells and those cells expressing EGFP or FUS-GFP-WT displayed little activation of CHOP, in contrast to cells expressing mutant FUS-GFP-R521G with empty vector. However, over-expression of PDI reduces nuclear CHOP immunoreactivity in mutant FUS-GFP-R521G cells. Scale bar = 5 µm. (**B**) Quantification of cells in (**A**). A significant reduction (****p < 0.0001) in the proportion of mutant FUS-GFP-R521G expressing cells with ER stress was detected following co-expression with PDI, compared to mutant FUS cells co-expressing vector only. Values represent mean ± SD, n = 3. ****p < 0.0001.

We next examined whether PDI is protective against ER stress induced by mutant FUS, which can be detected by nuclear immunoreactivity to CHOP, a transcription factor induced during the UPR^20^. CHOP translocates to the nucleus during ER stress, hence nuclear immunoreactivity to CHOP can be used to detect UPR induction in cells expressing mutant FUS^20^. Neuro-2a cells were transfected as above and immunocytochemistry was performed using anti-CHOP antibodies. Immunoreactivity in the nucleus was then quantified (Fig. 5A). Only a small proportion of untransfected cells (Un), EGFP only or FUS-GFP-WT expressing cells (7%, 4% and 5% respectively) displayed nuclear CHOP immunoreactivity, indicating induction of the UPR. As previous^20^, a significantly greater proportion of FUS-GFP-R521G expressed with empty vector cells displayed ER stress in the absence of PDI compared to FUS-GFP-WT cells (22%, ****p < 0.0001). However, when PDI was co-expressed with mutant FUS, this proportion was significantly reduced to 8% (****p < 0.0001, Fig. 5B). Hence, over-expression of PDI inhibits ER stress induced by mutant FUS R521G.

### Over-expression of PDI is protective against mutant FUS induced apoptosis in a neuronal cell line and primary cultures

**Figure 6:**
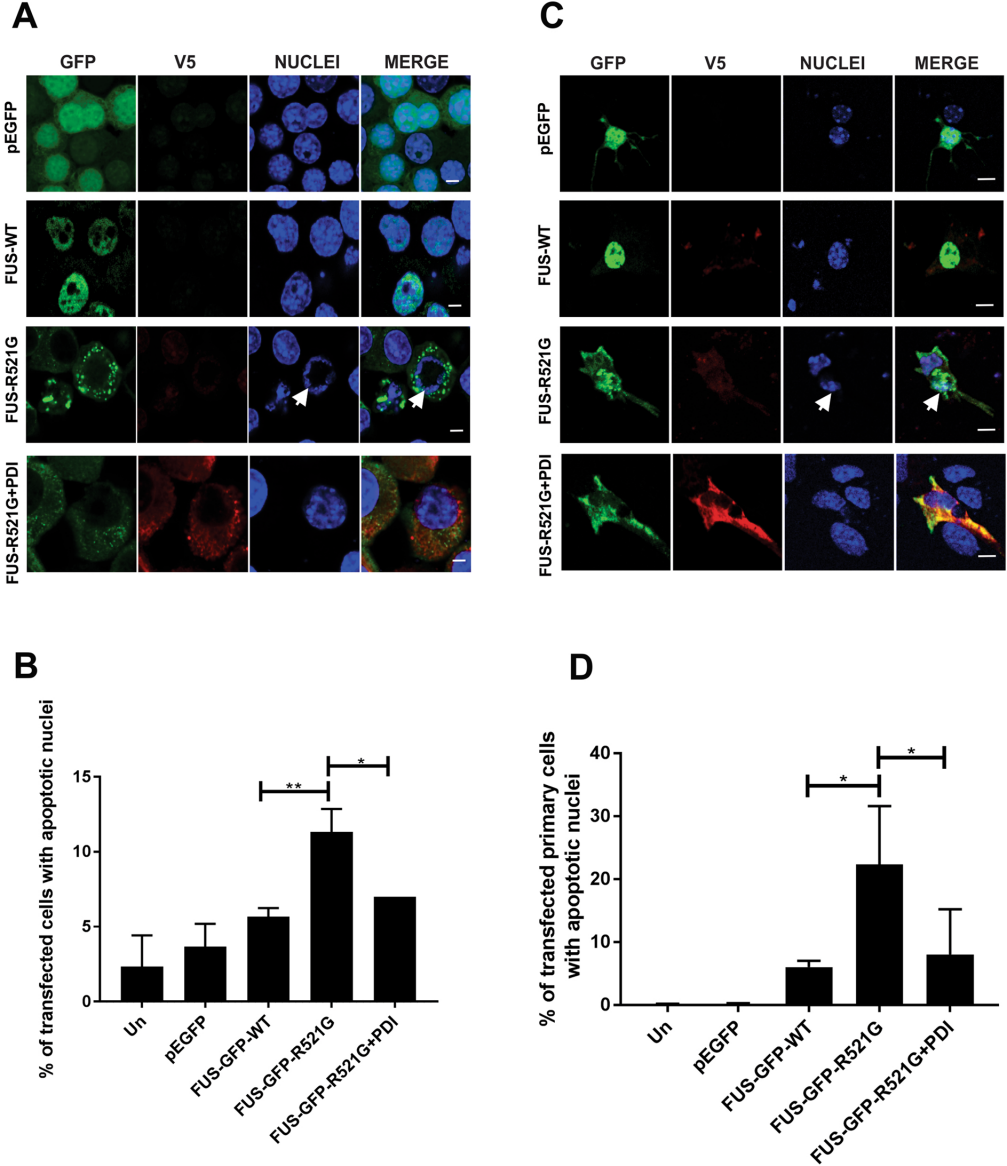
Over-expression of PDI is protective against mutant FUS-induced apoptosis in a neuronal cell line and primary cultures. (**A**) Neuro-2a cells co-expressing EGFP, FUS-GFP-WT or FUS-GFP-R521G with PDI, were examined by confocal microscopy at 72 h post-transfection following nuclei staining using Hoechst 33342 (blue). White arrows represent condensed or fragmented nuclei, indicating that apoptosis is underway. Few untransfected cells or those expressing EGFP (row 1) or FUS-GFP-WT (row 2) contained fragmented nuclei (< 5%), but more cells expressing FUS-GFP-R521G with empty vector (row 3) displayed Hoechst-stained condensed nuclei, indicating induction of apoptosis. Fewer cells co-expressing FUS-GFP-R521G with PDI (row 4) were undergoing apoptosis compared to those co-expressing mutant FUS with empty vector. Scale bar = 5 µm. (**B**) Quantification of apoptotic nuclei in cells in (**A**) expressing FUS and PDI. Over-expression of PDI with FUS-GFP-R521G resulted in significantly fewer cells with apoptotic nuclei compared to cells transfected with empty vector only (*p < 0.05). A significant difference was also observed between FUS-GFP-WT and mutant FUS-GFP-R521G (**p < 0.01) expressing cells. (**C**) Mouse primary cells co-expressing EGFP only (row 1), FUS-GFP-WT (row 2) or FUS-GFP-R521G with empty vector (row 3), with PDI (rows 4) and counter staining with Hoechst 33342 stain to visualise apoptotic nuclei, indicated by white arrows. Scale bar = 10 µm. (**D**) Quantification of apoptotic nuclei in cells in (**C**) expressing FUS and PDI. Significantly fewer cells were apoptotic when PDI was co-expressed with FUS-R521G (*p < 0.05) compared to those expressing vector only. Values represent mean ± SD, n = 3 **p < 0.01, *p < 0.05.

Finally, we examined whether PDI is protective against apoptosis induced by mutant FUS-GFP-R521G. Neuro-2a cells co-expressing EGFP, FUS-GFP-WT or mutant FUS-GFP-R521G with either empty vector or PDI-V5 as above were counter-stained for Hoechst 33342. The presence of fragmented or condensed nuclei, visualized using Hoechst 33342 staining, is a reliable marker of apoptosis, as examined previously^24–26^ (Fig. 6A). Healthy cells were observed mostly as compact and round cells. In contrast, apoptotic cells displayed condensed (under ∼ 5 μm in diameter), or fragmented (many Hoechst-positive condensed structures in one cell) nuclei. Few cells expressing EGFP alone (4%) or FUS-GFP-WT (6%) displayed apoptotic nuclei, as expected, whereas 11% (**p < 0.01) of FUS-GFP-R521G cells co-expressing empty vector contained fragmented nuclei (Fig. 6B). However, when PDI was co-expressed with mutant FUS, significantly fewer cells possessed apoptotic nuclei compared to those expressing FUS-GFP-R521G with empty vector (7%, *p < 0.05). Hence, these results imply that PDI is protective against apoptosis triggered by mutant FUS-R521G.

To confirm these observations obtained in a cell line, primary mouse cortical cells were next co-transfected with FUS-GFP-WT, FUS-GFP-R521G and PDI-V5 as above (Fig. 6C). Few primary cells expressing FUS-GFP-WT (6%), EGFP or untransfected cells, bore apoptotic nuclei. This contrasted with 22% (*p < 0.05) of cells co-expressing mutant FUS-GFP-R521G with empty vector (pcDNA3.1). However, this proportion was significantly decreased to 8% (*p < 0.05) when PDI was co-expressed with mutant FUS (Fig. 6D). Hence these data confirm that PDI is protective against apoptosis induced by mutant FUS.

## Discussion

Mutations in FUS are present in 5% of all fALS cases^5,6^ and the histopathological hallmark of FUS-ALS is the formation of FUS-positive inclusions in motor neurons and glia^5,6,36^. Moreover, protein misfolding is a central mechanism in ALS pathogenesis. Hence mechanisms to prevent protein misfolding may have potential therapeutically. We recently reported that the PDI family of proteins protect against the misfolding of mutant SOD1 and mutant TDP-43^24^. Furthermore, novel roles for PDI proteins were recently identified in neurons in mediating motor function and neuronal connectivity^37,38^. However, PDI is aberrantly modified by S-nitrosylation in ALS patients^26^ which results in loss of its functional activity^24,26^ Interestingly, however, PDI colocalizes with FUS in human tissues from both sALS and fALS patients and in cells expressing mutant FUS^20^, implying that it may be protective against mutant FUS given its chaperone function. Similarly, PDI is also known to immunoprecipitate with FUS^20,21^. However, it remained unclear if PDI has a protective role against mutant FUS. In this study, we demonstrate that PDI was protective against multiple cellular defects induced by mutant FUS; inclusion formation, inhibition of both nuclear import and ER-Golgi transport, Golgi-fragmentation, ER stress and apoptosis, in a neuronal cell line and primary mixed neuron-glia cultures. Thus, this study implies that PDI may be a new therapeutic target against FUS-associated ALS.

An important finding of this study is the ability of PDI to restore nuclear import in cells expressing mutant FUS. ALS-associated FUS mutations in the NLS domain impair nuclear import^33^, resulting in the mislocalisation of FUS to the cytosol and the generation of stress granules (SGs), which are linked to inclusion formation^17–19^. Similarly, PDI was also protective against the formation of mutant FUS inclusions. Indeed, the nucleus is now implicated as an important site of toxicity relevant to ALS pathogenesis^14,39^. Disruption of nucleocytoplasmic transport is also induced by ALS-associated TDP-43^30^ and by C9orf72 mutations^39–41^. Together these studies highlight the importance of nucleocytoplasmic defects in ALS.

Although conventionally regarded as being ER-localised, it is now well established that PDI is also found in other cellular locations, including the nucleus and cytoplasm^42–44^. The protective effects of PDI against the inhibition of nucleocytoplasmic shuttling and FUS misfolding therefore imply that this is mediated from the cytoplasm. Interestingly, PDI redistributes away from the ER in punctate vesicles in ALS models, where it displays higher enzymatic activity^45,46^. Furthermore PDI inhibits ALS progression in mice in vivo when expressed in this location^45^. Moreover, a recent study demonstrated that PDI translocates away from the ER into the cytoplasm following the induction of ER stress^47^. Hence, in cells expressing mutant FUS, PDI may re-locate from the ER to the cytoplasm, from where it is protective. However, given that FUS has been detected within the ER^21^, a protective role for ER-localised PDI is also possible. Hence, further characterisation of the cellular location of PDI in cells expressing mutant FUS is warranted in future studies.

Golgi fragmentation is a well described phenomenon in ALS^48^ that is known to occur when protein export from the ER is altered, or when vesicular trafficking from either to or from the Golgi is perturbed^49^. The formation of Golgi stacks requires the continuous recycling of proteins from or to the ER, therefore bidirectional vesicular transport with the ER is essential for Golgi organization^50^. Misfolded and incompletely folded proteins are excluded from transport vesicles leaving ER exit sites during the first phases of ER-Golgi transport^49–53^. Inhibition of ER-Golgi traffic could therefore increase the load of proteins within the ER, inducing ER stress^21^. Mutant FUS inhibits ER-Golgi trafficking, which could thus explain both fragmentation of the Golgi as well as induction of ER stress^20^. However, mutant FUS within the ER may also induce ER stress^21^. Hence PDI may be protective by both restoring ER-Golgi transport and reducing the load of misfolded proteins within the ER.

We recently demonstrated that the oxidoreductase activity of PDI is necessary for its protective effects against mutant SOD1 and mutant TDP-43^24^. It remains unclear whether the chaperone or oxidoreductase activity of PDI is protective against mutant FUS. However, whilst aberrant disulphide bond formation between specific cysteine residues in both mutant SOD1 and mutant TDP-43 are implicated in protein misfolding^54,55^, no disulphide bonds have been identified as yet in FUS (native or otherwise), despite FUS containing 4 cysteines that are part of the zinc finger involved in RNA-binding^56^. FUS contains a prion-like domain and its ability to aggregate is determined by the presence of this domain^57^. Interestingly, PDI has been shown to interact with prion-like misfolded proteins, implying that PDI may interact with FUS by its prion domain^58^. Together, these studies imply that PDI is protective against mutant FUS by its chaperone, rather than its oxidoreductase activity, although further studies are required to examine this possibility.

PDI is S-nitrosylated in patients with ALS as well as in other neurodegenerative disorders^26^. This aberrant post-translational modification could therefore mask the normal protective function of PDI during disease. S-nitrosylation of PDI may compromise its protective functions, further aggravating protein misfolding and ER stress in these conditions. Protein misfolding could additionally further deplete PDI by sequestration, thus reducing the effective cellular pool of chaperones. This vicious cycle may trigger the onset of neurodegeneration due to the collapse of multiple cellular processes, thus dysregulating proteostasis. FUS is also present in the pathological inclusions of patients with FTD without TDP-43 or tau pathology, accounting for about 10% of FTLD cases. It is therefore tempting to speculate that PDI may also have a protective role against FTLD.

In summary, in this study it was demonstrated that PDI, a unique chaperone with diverse activities, is protective against important pathogenic mechanisms associated with cellular function and apoptosis in FUS-associated ALS. The findings of this study therefore provide further evidence that PDI may be an effective potential therapeutic target against multiple proteins and cellular pathologies in ALS.

## Methods

### Expression constructs

Wild-type FUS (FUS-WT) and mutant FUS (R521G) constructs encoding C-terminus GFP-tagged human FUS were provided by Prof Justin Yerbury, Wollongong, Australia^21,29^. FUS-WT and FUS-R521G constructs encoding hemagglutinin (HA)-tagged human FUS at the N-terminus were a kind gift from Dr. Dorothee Dormann, Munich University. VSVG^ts045^ tagged with mCherry was a generous gift from Dr Jennifer Lippincott-Schwartz and Dr. George Patterson, National Institutes of Health, USA. Previously generated pcDNA3.1(+) constructs encoding PDI were kindly provided by Professor, Neil Bulleid, University of Glasgow, UK^59^. A transport reporter plasmid encoding lentiviral-S-tdTomato (Std-Tomato) was a kind gift from Prof. Jeffrey Rothstein (Addgene plasmid #112579)^31^.

### Cell culture and transfection

Neuro-2a cells were maintained in DMEM with 10% FBS and incubated at 37 °C with 5% CO_2_. Transfections were performed using LipofectamineTM 2000 (Invitrogen) according to the manufacturer’s protocol. Cells were co-transfected with FUS (GFP or HA) and PDI-V5 constructs and observed 48 h or 72 h post-transfection using fluorescence microscopy, unless specified otherwise.

### Immunoblotting

Cell lysates were collected in cold TN buffer (50 mM Tris–HCl pH 7.5 and 150 mM NaCl, pH 7.6) with 0.1% [w/v] sodium dodecyl sulfate (SDS), 1% Triton-X100, 1% protease inhibitor cocktail (Roche) and 1% phosphatase inhibitor (Roche) and phenylmethanesulfonyl fluoride [PMSF, Sigma #P7626–250 MG], then incubated on ice for 15 min and stored at − 20 °C overnight. Samples were centrifuged at 100,000*g* at 4 °C for 30 min to obtain the SDS-soluble fraction. Protein concentrations of cell lysates were determined using the BCA protein assay (Thermo Scientific) by comparison with BSA standards. Protein samples (10–20 µg) were electrophoresed through 8.5% SDS–polyacrylamide gels and transferred to nitrocellulose membranes. Membranes were blocked with 5% skim-milk in Tris-buffered saline (pH 8.0) for 30 min, then incubated with the appropriate primary antibodies at 4 °C for 16 h; anti-FUS (1:500, Abcam ab23439), anti-V5 (1:1000, Invitrogen P/N460705), or anti-actin (1:1000, Cytoskeleton #AAN01). Membranes were incubated for 1 h at room temperature with secondary antibodies (1:2000, HRP-conjugated goat anti-rabbit, or goat anti-mouse, Merck Milipore, AP130, AP132), and detected using ECL reagent (Bio-Rad). Precision Plus Protein™ Dual Colour Standard molecular weight markers were used (Bio-Rad). Quantitation of blots was performed by densitometry using ImageJ (NIH).

### Immunofluorescence and microscopy

Neuro-2a cells were fixed in 4% paraformaldehyde, permeabilized with 0.1% Triton-X100 in phosphate buffered saline (PBS), blocked with 3% BSA in PBS, followed by incubation with mouse anti-CHOP (1:50, Santa Cruz Sc-7351), mouse anti-V5 (1:250, Invitrogen P/N460705), or mouse anti-GM130 (1:250, BD Transduction Lab 610823) antibodies in PBS at 4 °C overnight. The secondary antibody AlexaFluor 568-conjugated rabbit anti-mouse IgG (1:250, Invitrogen A21203) was added for 1 h and incubated in the dark at room temperature. After washing with PBS, staining of nuclei was performed using Hoechst stain 33342 (Invitrogen, nuclei). FITC (for GFP fluorescence) and TRITC (for red fluorescence) filters were used for viewing the cells and images were taken using a Zeiss Axioimager microscope or LSM 880 Zeiss confocal microscope in a single focal plane. In dual-channel imaging, photomultiplier sensitivities and offsets were set to a level at which bleed-through effects from one channel to another were negligible. Nuclear and cytoplasmic fluorescent intensity of either the Std-Tomato reporter or FUS and PDI was calculated with the same exposition time between groups, using ImageJ.

For Fig. 2, the pharmacological agent Mifepristone (Cayman Chemical #10006317) was used as a positive control. The corrected total cell fluorescence (CTCF) of the whole cell and the nucleus was determined and then the ratio was obtained by dividing the CTCF of Std-tomato expression in the nucleus by that of the whole cell using Image J software^60^. In brief, 20 cells were scored for each experiment and cells of interest were selected using drawing/selection tools (freeform) from Image J. From the ‘Analyze’ menu, “Set measurements” was selected by marking AREA, INTEGRATED DENSITY and MEAN GRAY VALUE. The cell fluorescence was measured in the nucleus and then the whole cell by selecting “Measure” from the ‘Analyze’ menu. A region next to the cell lacking fluorescence was measured as background. The formula used was as follows; (CTCF = Integrated Density – (Area of selected cell × Mean fluorescence of background readings).

### Cortical cell culture and transfection

Primary cultures were harvested from the cortex of C57BL/6 mouse embryos at embryonic day 16–18. The procedure for culture of primary cells was as described previously^25^. Briefly, cortical tissue was dissected under sterile conditions in Hanks’ Balanced Salt solution (HBSS, Gibco) and digested in 10 units/ml papain (Sigma) in 2 mg/ml l-cysteine (Sigma) and 0.5 mM EDTA, pH 8 (Sigma) in DMEM, for 15 min at 37 °C. Cells were subsequently triturated using pipette tips so they were dissociated, then resuspended in plating medium (DMEM, 10% FBS, 100 µg/ml penicillin–streptomycin) and seeded for 1 h on 15 mm glass coverslips, previously coated with 0.1 mg/ml poly-d-lysine overnight. Cells were then incubated in neuronal medium [Neurobasal medium supplemented with 2% B27, supplement (Gibco), 1% Glutamax (Gibco) and 100 µg/ml penicillin–streptomycin] at 37 °C and 5% CO_2_.

For primary cultures, after 5–7 days in vitro, cultured cortical cells were co-transfected for 48 h with 0.5 µg of pEGFP-N1 vector, GFP-tagged FUS-WT or mutant FUS-R521G with 0.5 µg of plasmid encoding V5-tagged PDI or empty vector pcDNA3.1, using 2 µl LipofectamineTM 2000 (Invitrogen), following the manufacturer’s instructions.

### Primary cell immunocytochemistry

Cortical primary cells transfected with FUS constructs for 48 h were washed in PBS and fixed in 4% paraformaldehyde in PBS for 10 min. After three washes in PBS, cells were permeabilized in 0.1% Triton-X100 in PBS for 5 min and the non-specific background staining was blocked using 3% (wt/v) BSA in PBS for 45 min at room temperature with gentle rocking. Cells were then incubated overnight at 4 °C with anti-V5 antibody (1:250, Invitrogen P/N460705) in 3% (wt/v) BSA in PBS. After rinsing, cells were incubated for 1 h at room temperature with anti-mouse secondary antibody coupled to AlexaFluor 594 (Invitrogen A21203), diluted 1:250 in PBS. Cells were then washed as above and treated with 0.5 µg/ml Hoechst 33342 reagent (Sigma). After washes in PBS, coverslips were mounted onto slides in fluorescent mounting medium (Dako). At least 20 cells were then examined and photographed on a Zeiss LSM 880 inverted confocal laser-scanning microscope, equipped with a LSM-TPMT camera (Zeiss).

### VSVG^ts045^ ER-Golgi transport assay

Neuro-2a cells were transiently transfected with the appropriate construct and the VSVG^ts045^-mCherry plasmid. Cells were incubated at 40 °C under 5% CO_2_ for 72 h with FUS. The cells were then treated with cDMEM containing cycloheximide (20 μg/ml) and incubated at 32 °C for 30 min. Staining was performed with primary antibodies mouse, anti-GM130 (1:250) (Golgi marker) (BD Transduction 610823) or rabbit anti-calnexin (1:250) (ER marker) (Abcam ab22595) overnight. Secondary antibodies AlexaFluor 647 goat anti-mouse (1:200 Life Technology A21235) and goat anti-rabbit (1:200 Thermo Fisher Sci A27040) were used. Mander’s coefficient was used to determine the degree of co-localisation between VSVG^ts045^-mCherry and ER or Golgi marker. Mander’s coefficient was calculated for 20 cells by JACoP (Colocalisation Plugin)^61^ in ImageJ (NIH).

### Statistical analysis

The experiments were performed a minimum three times on separate days with one blinded experiment, unless specified separately. The data are represented as mean ± SD. One-way Analysis of Variance (1-way ANOVA) followed by Tukey post hoc test was used to determine between treatment differences (GraphPad Prism, San Diego, CA, USA). A p-value of 0.05 or equal was considered significant, where ****p < 0.0001, ***p < 0.001, **p < 0.01, *p < 0.05. 100 cells were scored for each population unless otherwise stated.

## Supplementary Information

Supplementary Information.
