# Supplementary material for: Protein disulphide isomerase (PDI) is protective against amyotrophic lateral sclerosis (ALS)-related mutant Fused in Sarcoma (FUS) in in vitro models
Source: Sci Rep. 2021 Sep 2;11:17557. doi: 10.1038/s41598-021-96181-2 (PMC8413276; doi:10.1038/s41598-021-96181-2)

Supplementary figure

Protein disulphide isomerase (PDI) is protective against amyotrophic lateral sclerosis (ALS)-related mutant Fused in Sarcoma (FUS) in in vitro models.

S Parakh1,3, E R. Perri1,3, M Vidal1, J Sultana1,3, S Shadfar1, P Mehta1, A Konopka1, C J. Thomas2, D M. Spencer3 and J D. Atkin1,3

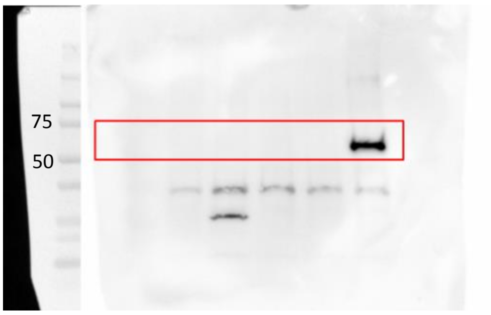

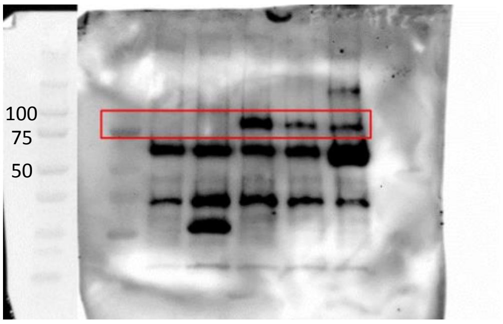

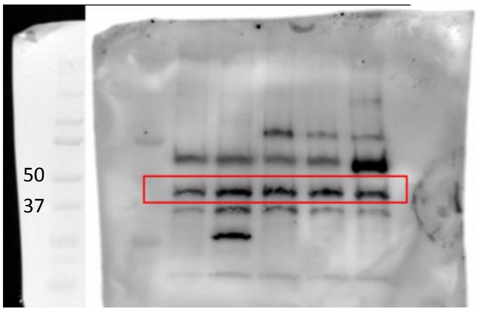

Supplement: Supplementary file 1 — Supplementary Information. [file 41598_2021_96181_MOESM1_ESM.pdf]
